# NFATc1 phosphorylation by DYRK1A increases its protein stability

**DOI:** 10.1371/journal.pone.0172985

**Published:** 2017-02-24

**Authors:** Heng Liu, Ketao Wang, Shuai Chen, Qian Sun, Yuankai Zhang, Long Chen, Xiulian Sun

**Affiliations:** 1 Shenzhen Research Institute of Shandong University, Shenzhen, Guangdong Province, China; 2 Otolaryngology Key Lab of Ministry of Health, Qilu Hospital of Shandong University, Jinan, Shandong Province, China; 3 Brain Research Institute, Qilu Hospital of Shandong University, Jinan, Shandong Province, China; Charles P. Darby Children's Research Institute, 173 Ashley Avenue, Charleston, SC 29425, USA, UNITED STATES

## Abstract

NFATs are transcription factors involved in immune activation and tumor progression. Previous reports showed that DYRK1A suppressed NFATc2 transcriptional activity through phosphorylation. Nonetheless, our results showed that DYRK1A increased NFATc1/α*A* protein level and subsequent transcriptional activity. DYRK1A phosphorylation of NFATc1/α*A* at S261, S278, S403 and S409 interfered with NFATc1 ubiquitination and ubiquitin-proteasome degradation. Our results imply that DYRK1A is a positive kinase in regulation of NFATc1.

## Introduction

Nuclear factor of activated T-cells (NFAT) transcription factor family consists of five members. Though originally characterized in immune cells, NFATs are ubiquitously expressed, and most at least one family member in different cell types [1, 2]. The NFATc1-c4 can be regulated by calcium-calcineurin and contains two homology domains: the Rel homology region (RHR) and NFAT homology region (NHR) [3]. Moderately conserved NHR contains the transactivation domain, in which numerous serine/threonine residues are phosphorylated by various kinases in resting cells. Upon calcium influx, dephosphorylation of these residues by calcineurin will expose the nuclear localization signal and translocate NFAT into nucleus, where it binds to promoter elements and initiates transcription [1]. Dual specificity tyrosine-phosphorylation regulated kinase 1 (DYRK1) and DYRK2 phosphorylate NFATc2 on the serine-proline repeat 3 (SP3) motif, thus priming the subsequent phosphorylation of the SP2 and SRR1 motifs by Casein kinase 1 and glycogen synthase kinase 3 (GSK-3). DYRK1 functions as an NFATc2 export kinase, whereas DYRK2 phosphorylates NFATc2 in the cytoplasm and functions as a maintenance kinase [4, 5]. DYRK1A, located within the HSA21q22.2, is the mammalian homologue of the *Drosophila minibrain* gene [6–8]. By phosphorylating the SP motif of NFAT, DYRK1A can cooperate with regulator of calcineurin 1 (RCAN1) and counteract with calcineurin in regulation of NFAT signaling pathways [4, 5]. DYRK1A decreased transcriptional activity of NFATc2, NFATc3 and NFATc4; however, it is unknown how DYRK1A phosphorylates NFATc1 and affects its activity. Because of the existence of two promoters, two poly A sites and alternate splicing events, six NFATc1 proteins are expressed in peripheral lymphocytes with different deletions of C-termini. Study showed functional differences between NFATc1 and NFATc2 might be due to the synthesis of NFATc1/α*A*, a short isoform of NFATc1, which lacks the C-terminal peptide of approximately 250 amino acid residues typical for most of the other NFATc proteins. Only this NFATc1/α*A* isoform was shown to exert oncogenic effects, while the longer one of NFATc1 resembles the functions of NFATc2 [9]. Here, we demonstrated that DYRK1A increased NFATc1 (NFATc1/α*A* isoform) protein stability, in contrast to the decrease of NFATc2 protein stability by DYRK1A. The phosphorylation of NFATc1 by DYRK1A reduced the subsequent ubiquitination, resulting in increased NFATc1 protein stability. The distinct effects of DYRK1A on NFATc1 from NFATc2 further indicate distinct role of NFATc1 in immune system and tumorigenesis.

## Results

### DYRK1A increased NFATc1 protein expression and its nuclear translocation

**Fig 1:**
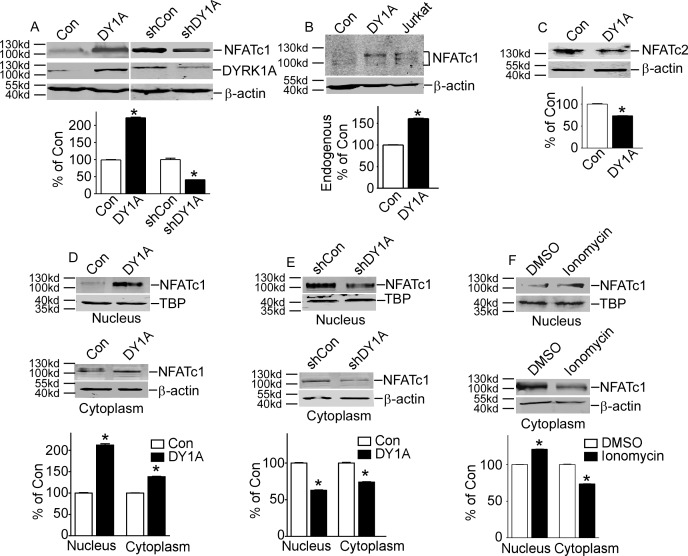
DYRK1A increased NFATc1 protein expression and its nuclear translocation. **A.** HEK293 cells were transfected with p-NFATc1mycFLAG and pCMV-DYRK1A or pGFP-V-RS-shDYRK1A. NFATc1 expression was detected by mouse anti-FLAG antibody immunoblotting. DYRK1A was blotted with anti-DYRK1A antibody. **B.** Endogenous NFATc1 was detected by anti-NFATc1 antibody in HEK293 cells transfected with pCMV-DYRK1A. An activated Jurkat T cells treated with 5uM ionomycin plus 500uM calcium chloride for 12 hours was used as positive control. **C.** HEK293 cells were co-transfected with pWT-NFATc2 and pCMV-DYRK1A. Anti-HA antibody was used to detect NFATc2. **D and E.** HEK293 cells were co-transfected with p-NFATc1mycFLAG and pCMV-DYRK1A orpGFP-V-RS-shDYRK1A. Mouse anti-FLAG antibody was used to detect NFATc1 protein in nucleus or cytoplasm.Β-actin was used as cytoplasmic control and TBP was used as nuclear protein loading control. **F.** HEK293 cells were treated with ionomycin. Mouse anti-FLAG antibody was used to detect NFATc1 protein in nucleus or cytoplasm β-actin was used as cytoplasmic control and TBP was used as nuclear protein loading control. Values represent means±SD, n = 3. DY1A means DYRK1A. shDY1A means shDYRK1A.

To examine whether NFATc1 protein level was affected by DYRK1A, pNFATc1mycFLAG was co-transfected with pCMV-DYRK1A or pGFP-V-RS-shDYRK1A into HEK293 cells. The pGFP-V-RS-shDYRK1A can specifically knock down DYRK1A expression [10]. The protein level of NFATc1 was increased to 222.20%±0.02 with DYRK1A over-expression (P<0.001, lane 2 compared to lane 1 of Fig 1A). And NFATc1 protein expression was decreased to 41.13%±0.01 with DYRK1A knockdown (P<0.001, lane 4 compared to lane 3 of Fig 1A). Over-expression and knockdown of DYRK1A were also detected (Fig 1A middle lane) Consistent with this, endogenous NFATc1 protein expression was also increased to 122.50%±0.02 by DYRK1A, and an activated T cells treated with ionomycin was used as positive control which showed a mixture of long and short isoforms of NFATc1 (P<0.001, Fig 1B). Consistent with previous reports, over-expression of DYRK1A markedly decreased exogenous NFATc2 protein level to 41.13%±0.01 (P<0.001, Fig 1C).

Activation of NFATc1 requires its nuclear translocation. To investigate whether protein increase of NFATc1 also results in increased protein in nucleus, HEK293 cells co-transfected with p NFATc1mycFLAG and pCMV-DYRK1A were fractionated into cytoplasm and nucleus. The results showed nucleus and cytoplasm NFATc1 were increased to 212.2±3.2% (P<0.001) and 138.2±0.75% (P<0.001) with over-expression DYRK1A, respectively (Fig 1D). On the other hand, DYRK1A knock-down decreased cytoplasm and nucleus NFATc1 to 63.0±0.45% (P<0.001) and 74.0±0.52% (P<0.001) (Fig 1E). HEK293 cells transfected with p NFATc1mycFLAG were further activated with ionomycin. The results showed that nucleus NFATc1 was increased to 123.2±0.02% (P<0.001) by ionomycin, but cytoplasm NFATc1 was deceased to 71.1±0.02% (P<0.001), indicating ionomycin translocated NFATc1 from cytosol into nucleus (Fig 1F). These data showed that NFATc1 protein expression was increased in cytoplasm and nucleus by DYRK1A.

### DYRK1A affected NFATc2 protein ubiquitination and stability through phosphorylation

**Fig 2:**
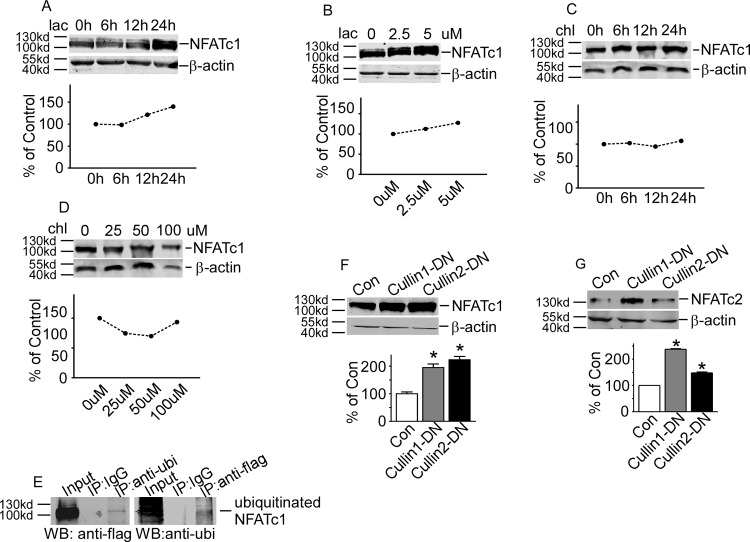
DYRK1A affected NFATc2 protein ubiquitination and stability. **A and B**. HEK293 cells transfected with p-NFATc1mycFLAG were treated with proteasome inhibitorlactacystin (lac) for time or dosage indicated. **C and D**. HEK293 cells transfected with p-NFATc1mycFLAG were treated with lysosome inhibitor chloroquine (chl) for time or dosage indicated. **E.** Co-IP assay showed the cross-interaction between NFATc1 and ubiquitin. **F and G.** NFATc1 and NFATc2 were increased by inhibition of SCF E3 ligase using Cullin-1/2 DN constructs. Values represent means±SD, n = 3.Lacmeanslactacystin. Ubi means ubiqutin.

To examine whether the increased expression of NFATc1 by DYRK1A is associated with its protein stability, HEK293 cells transfected with p-NFATc1mycFLAG were exposed to lactacystin, a specific proteasome inhibitor. The NFATc1 protein was markedly increased in a time-dependent manner and a dosage-dependent manner by inhibition of proteasome (Fig 2A and 2B). The autophagy-lysosome pathway inhibitor chloroquine did not affect the NFATc1 protein degradation (Fig 2C and 2D). These data implied that NFATc1 could be degraded by ubiquitin-proteasome pathway. To investigate whether NFATc1 was ubiquitinated, co-immunoprecipitation assay was applied in HEK293 cells co-transfected with p- NFATc1mycFLAG and pHis-ubi. Anti-FLAG antibody detected a band in anti-ubiquitin immunoprecipitates (Fig 2E, lane 3) that migrated together with NFATc1 protein in the cell lysate (Fig 2E, lane 1). And vice versa, the anti-ubiquitin antibody detected the NFATc1 protein pulled down with anti-FLAG antibody (Fig 2E, lane 6). These results indicate that NFATc1 degradation was mediated by the ubiquitin-proteasome pathway.

Protein ubiquitination is mediated by three components, including ubiquitin-activating enzyme (E1), ubiquitin conjugating enzyme (E2) and ubiquitin ligase (E3). The SCF complex is an important E3 ubiquitin ligase, consisting skp1, Cullins, F-box protein and RBX1. To further define the E3 ligase mediating the NFATc1 ubiquitination, HEK293 cells were co-transfected with p- NFATc1mycFLAG and the Cullin1 and Cullin2 domain-negative constructs[11]. Inhibition of E3 ligase activity by Cullin1-DN and Cullin2-DN increased NFATc1 protein levels to 194.7%±13.05 (P<0.01) and 223.0%±11.81 (P<0.001) (Fig 2F). Also, inhibition of E3 ligase increased exogenous NFATc2 protein levels to 237.7.7%±0.02 (P<0.01) and 148.0%±0.03 (P<0.001) (Fig 2G). These results further confirmed that both of NFATc1 and NFATc2 may be degraded through SCF E3 ligase mediated ubiquitin-proteasome pathway.

**Fig 3:**
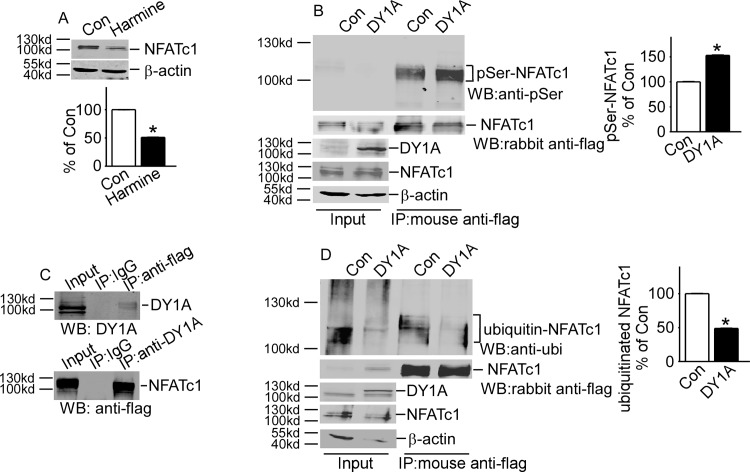
DYRK1A affected NFATc2 protein phosphorylation. **A.** DYRK1A inhibitor harmine (2ug/ml) decreased NFATc1 expression (the image was flipped horizontally from the original picture). **B.** Anti-pSer antibody was used to detect the serine phosphorylated NFATc1 and rabbit anti-FLAG antibody was used to detect the total NFATc1 in the mouse anti-FLAG antibody immunoprecipitates. Mouse anti-DYRK1A antibody and mouse anti-NFATc1 antibody were used to detect total DYRK1A and total NFATc1, respectively in transfected cells. **C.** Co-IP assay showed the cross-interaction between NFATc1 and DYRK1A. **D.** Anti-ubiquitin antibody was used to detect the ubiquitination of NFATc1 and rabbit anti-FLAG antibody was used to detect the total NFATc1 in mouse anti-FLAG antibody immunoprecipitates. Mouse anti-DYRK1A antibody and mouse anti-NFATc1 antibody were used to detect total DYRK1A and total NFATc1, respectively in transfected cells, Values represent means±SD, n = 3. Ubi means ubiqutin. pSer means phosphorylation of serine.

Since DYRK1A is a kinase, we hypothesized that DYRK1A might affect NFATc1 protein ubiquitination and stability by phosphorylation of NFATc1. Consistent with this hypothesis, HEK293 cells were co-transfected with p-NFATc1mycFLAG, DYRK1A inhibitor harmine reduced NFATc1 protein to 50.88%±0.01 (p<0.001, Fig 3A). To investigate whether DYRK1A phosphorylates NFATc1, NFATc1 protein was pulled down using mouse anti-FLAG antibody in HEK293 cells co-transfected with p-NFATc1mycFLAG and pCMV-DYRK1A. NFATc1 protein phosphorylation was detected with serine phosphorylation antibody anti-pSer and total protein of NFATc1 was detected with rabbit anti-FLAG antibody. Serine phosphorylated NFATc1 protein was markedly increased to 150.90%±0.001 in presence of DYRK1A (p<0.001, Fig 3B, lane 4 compared to lane 3). Furthermore, HEK293 cells co-transfected with pNFATc1mycFLAG and pCMV-DYRK1A, co-IP assay showed that DYRK1A physically interacted with NFATc1 (Fig 3C). These data indicated that DYRK1A could phosphorylate NFATc1. To further investigate whether NFATc1 phosphorylation by DYRK1A would affect NFATc1 ubiquitination, NFATc1 protein was pulled down using mouse anti-FLAG antibody in HEK293 cells co-transfected with p-NFATc1mycFLAG and pCMV-DYRK1A. Total protein of NFATc1 was detected with rabbit anti-FLAG antibody, western blot using anti-ubiquitin antibody showed that NFATc1 ubiquitination was decreased to 56.8%±0.005 by DYRK1A (p<0.001, Fig 3D, lane 4 compared to lane 3), suggesting that DYRK1A regulated NFATc1 protein ubiquitination and degradation through phosphorylation.

### Identification of DYRK1A-targeted motifs in NFATc1 protein

**Fig 4:**
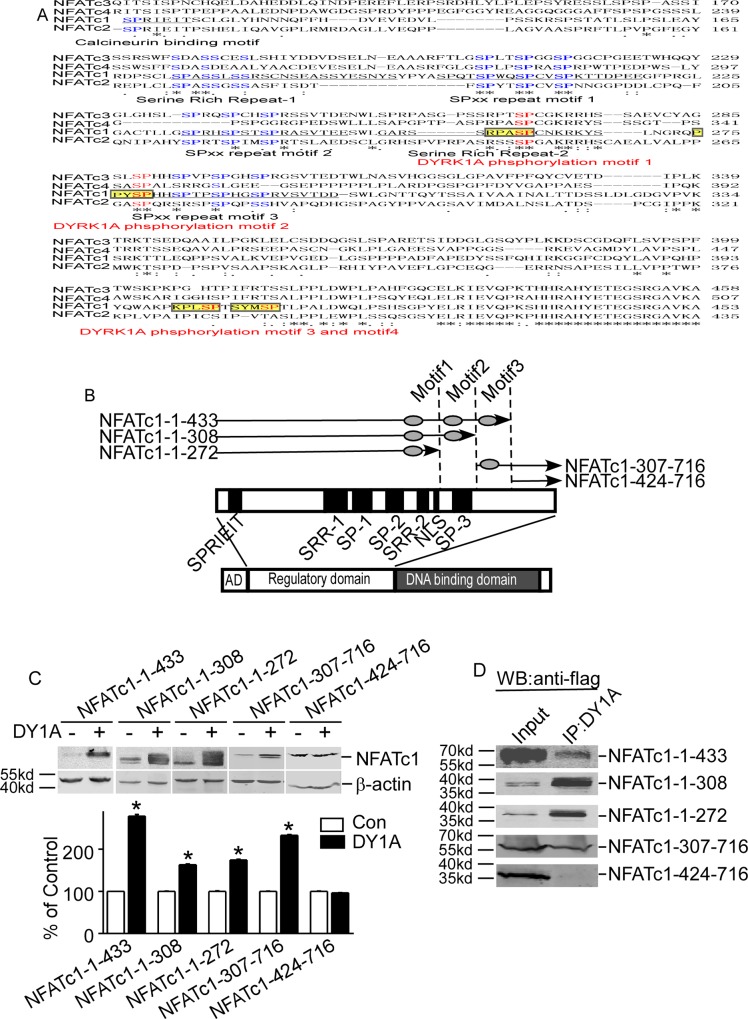
Identification of DYRK1A-targeted motifs in NFATc1 protein. **A.** Diagram showed NFATs sequence alignment. Sequences of NFATc1 (NP_765978), NFATc2 (NP_775114), NFATc3 (NP_775118), and NFATc4 (NP_001129494) were aligned using ClustalW2 software. Conserved regions among NFATs are underlined. Conserved serine residues in four NFATs are shown in blue. The DYRK1A target motifs are labeled as yellow highlights. The DYRK1A phosphorylation serine residues were in red and bold. **B.** Schematic diagram represents the domain structure of NFATc1 and location of five deletion constructs. AD: activation domain. SPRIEIT: calcineurin binding motif. SP: sp repeats region; SRR: serine-rich region; NLS: nuclear localization sequence. **C.** Five deletion constructs of NFATc1 were co-transfected with pCMV-DYRK1A in HEK293 cells. **D.** Deletionconstructs of NFATc1 was co-transfected with pCMV-DYRK1A in HEK293 cells. Anti-DYRK1A antibody was used as the immunoprecipitation antibody, and anti-FLAG antibody was used to detect NFATc1 in immunoblotting.

The substrates of DYRK1A contain a consensus RPX(S/T)P motif [12, 13]. Sequence alignment with RPX(S/T)P using ClustalW2 alignment tool identified four putative motifs in the regulatory domains of NFATc1 protein (Fig 4A). To further investigate which putative motifs were targeted by DYRK1A, five deleted constructs that contain different putative motifs were constructed (Fig 4B). The results showed that the over-expression of DYRK1A inhibited the degradation of four truncated mutants: NFATc1-1-433, NFATc1-1-308, NFATc1-1-272 and NFATc1-307-716 (Fig 4C). However, DYRK1A had no effects on deleted construct of NFATc1-424-716 that does not contain any putative motifs (Fig 4C). Co-IP assay showed that NFATc1-424-716 that did not contain any DYRK1A targeting motifs was the only construct that did not interact with DYRK1A (Fig 4D). This result indicated that the third and fourth motifs could be the DYRK1A targeting motifs.

**Fig 5:**
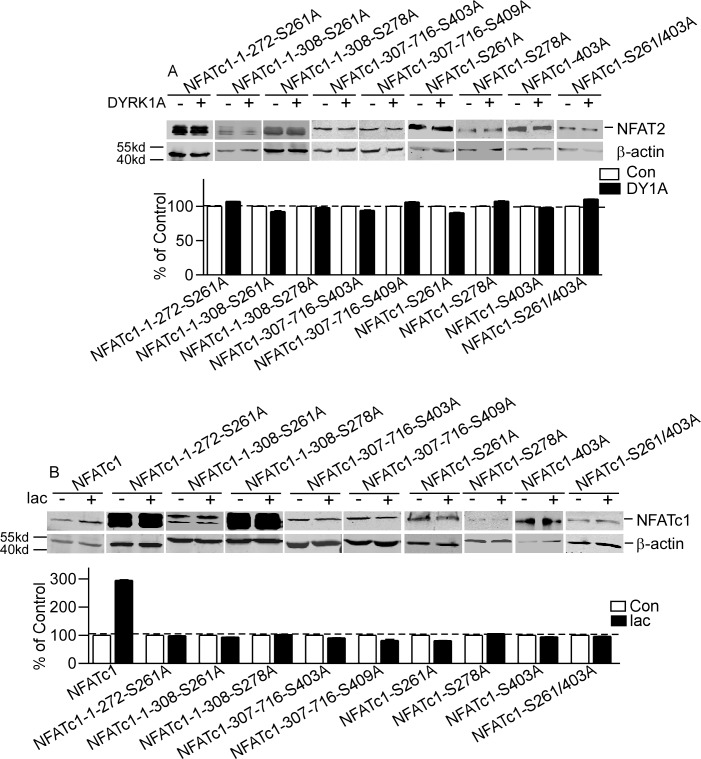
NFATc1 mutants abolished the effects of DYRK1A. **A.** Nine mutant constructs of NFATc1 were co-transfected with pCMV-DYRK1A in HEK293 cells. **B.** HEK293 cells co-transfected with NFATc1 and nine mutant constructs of NFATc1 were treated with proteasome inhibitor lactacystin (5uM) for 24 hours after transfection. Values represent means±SD, n = 3. DY1A means DYRK1A. ubi means ubiqutin. pSer means phosphorylated serine.

To further confirm the targeting motifs of DYRK1A, four serine residues S261, S278, S403 and S409 in the four motifs were mutated to alanines in full and deleted constructs. The results showed that DYRK1A had no effects on the mutant constructs (Fig 5A). Inhibition of proteasome pathway by lactacyctin did not affect protein levels of these mutant constructs, though lactacystin greatly increased protein levels of NFATc1 to 294.6±2.73% (P<0.001, Fig 5B). These results indicate that S261, S278, S403 and S409 were DYRK1A-targeted motifs.

## Discussion

Serfling E group indicated that functional differences between NFATc1 and NFATc2 might be due to the synthesis of NFATc1/α*A*, a short isoform of NFATc1 [9]. NFATc1/α*A*, which lacked the C-terminal peptide of approximately 250 aa typical for most of the other NFATc1 and NFATc2, was the most prominent NFAT protein in effector B cells and did not support activation-induced cell death as do other NFATs, which suggested that this C-terminal peptide plays an important role in inducing apoptosis relevant genes [9]. Only this NFATc1/α*A* isoform was shown to exert oncogenic effects, while the longer one of NFATc1 resembles NFATc2. The differences in oncogenic ability between NFATc1/α*A* and NFATc2 could be attributed to the C-terminal peptide of NFATc2, since removal of the 250 aa long C-terminal peptide from NFATc2 (including its SUMO sites) released its tumor suppressor phenotype and “converted” NFATc2 to a NFATc1/α*A*-like protein [14].

DYRK1A decreased NFATc2 stability while increased NFATc1 protein stability, suggesting that the same kinase may target various NFAT members and result in distinct functions. NFATc1 and NFATc2 show 72% homology in the DNA binding domain at the C-terminal (Fig 4A), but the function of these two subtypes is different [15]. In immune system, NFATc2 promotes Th1 cell differentiation and acts as a negative regulator of Th2 cell differentiation, whereas NFATc1 is required for Th2 cell differentiation in immune cells [3]. Th1 cells are characterized by the expression of IFN γ (interferon γ) and participate in the clearance of intracellular pathogens and also contribute to inflammation and autoimmune response. Th2 cells expressing IL-4, -5, and -13 constitute a defense against extracellular pathogens and are involved in atrophy and asthma [3]. The contradictory effects of DYRK1A on NFATc1 and NFATc2 interestingly reconciled to one same result: shifting the Th1 and Th2 ratio to Th2 dominant. NFATc1 has oncogenic activity, whereas NFATc2 acts as a tumor suppressor [3]. In addition to tumorigenesis, recent studies have shown that NFAT also promoted the invasive migration of tumor cells. Activation of NFATc1 is observed in some carcinomas such as Burkitt’s lymphoma and pancreatic cancer [3]. Both NFATc2 and NFAT5 promote the migration and invasion of breast and colon cancer cells [3]. So, the effect on tumorigenesis and cancer migration likely depended on the differential activation of NFATc1 and NFATc2 in different cancers. The distinct effects of DYRK1A on NFATc1 and NFATc2 further indicate distinct roles of NFATc1 and NFATc2 in immune system and tumorigenesis.

Yoeli-Lerner et al. have reported that NFATc2 was ubiquitinated by the E3 ligase murine double minute 2 downstream of Protein Kinase B and GSK-3 signaling in breast cancer cells [16, 17]. SCF complex compounds is the most important E3 ligase and ubiquitination mediated by SCF needed phosphorylation of substrates. Our results here showed that NFATc1 were degraded by ubiquitin-proteasome pathway. Ubiquitination of NFATc1 can be mediated by SCF E3 ligase downstream of DYRK1A phosphorylation of NFATc1 It is interesting to note that phosphorylation of NFATc2 by Akt, GSK-3 and DYRK1A lead to increased ubiquitination and protein instability, whereas phosphorylation of NFATc1 by DYRK1A inhibited ubiquitination and increased NFATc1 protein stability. Whether other NFAT family members are ubiquitinated and how their ubiquitinations are affected by phosphorylation remain to be determined. DYRK1A knockdown decreased NFATc1 stability (Fig 1A). Similarly DYRK1A inhibitor, the β-carboline alkaloid known as harmine that has been shown to bind to the ATP pocket of DYRK1A and inhibit its kinase activity [5], also decreased NFATc1 (Fig 3A). The SRR and the SP motifs were the usual targets of protein kinases. It was reported that the DYRK1A phosphorylation targets of NFATc2 were located in the SP3 motif [5]. Our study identified DYRK1A-target motifs at S261, S278, S403 and S409 of NFATc1. S261 and S278 are in conserved regions of SRR2 and SP3, whereas S403 and S409 have little conservation among NFATc1-c4 (Fig 4A). DYRK1A phosphorylated these serines of NFATc1 protein, which subsequently inhibited the protein ubiquitination and proteasome degradation. Meanwhile, results indicated that only one mutation of these serines could inhibit protein degradation, suggesting conformational changes caused by DYRK1A phosphorylation might influent the subsequent ubiquitination and proteasome degradation. Among the numerous substrates of DYRK1A, p27(Kip1)[5], p120-catenin[5], EGFR[5] and HPV16E7[5] was shown to be stabilized by DYRK1A phosphorylation, while cyclin D1[5], c-Myc[5] were destabilized upon phosphorylation by DYRK1A. Previous studies showed that phosphorylation by DYRK1A on NFATc2 [5] and NFATc4 [5] SP motifs could suppress nuclear translocation, followed by attenuated transcriptional activity. Nonetheless, these researchers did not find significant changes of total NFAT protein level. Here we found over-expression of DYRK1A could also cause increased phosphorylation of NFATc1 on the SP motifs. Furthermore, these phosphorylations could stabilize NFATc1 protein. DYRK1A in the nucleus phosphorylated NFATc1 and made NFATc1 turn out of nucleus. But E3 ligase couldn’t link ubiquitin to NFATc1, because of spatial conformation of phosphorylated NFATc1 changed, and lead to NFATc1 ubiquitin levels decreased. NFATc1 was not degraded by cytoplasmic proteasome and accumulated in cytoplasm. Calcineurin in cytoplasm continued to dephosphorylate NFATc1, then many dephosphorylated NFATc1 turned into the nucleus agnain and the relative amount of protein increased. Taken together, these studies indicated that DYRK1A regulated the activity of NFAT family members by different molecular mechanisms, resulting distinct effects on NFAT pathway.

## Materials and methods

### Cell culture and reagents

HEK293 cells purchased from ATCC (NumberCRL-1573™) were cultured at 37°C in an incubator containing 5% CO_2_ as previously described [10]. Cells were transfected with lipofactamine2000 (11668–027; Invitrogen, Carlsbad, CA, USA) according to the manufacturer’s guidelines. Lactacystin (426100) was purchased from Calbiochem (La Jolla, CA, USA). Chloroquinediphosphate salt (C6628), and harmine (286044) were purchased from Sigma-Aldrich (Louis, MO, USA).

### Plasmids construction

pWT-NFATc2 was from Addgene. The pCMV-DYRK1A, pGFP-V-RS-shDYRK1A and pHis-ubi were constructed in our lab. The cDNA of NFATc1 were amplified with (5’-cgcggatccgccacc ATGCCAAGCACCAGCTTT-3’) and (5’-ccgctcgag GAAAAAGCACCCCACGC -3’) and cloned into pCMV6-mycFLAG vector to make p- NFATc1mycFLAG. This NFATc1 cloned and used throughout this study was the isoform NFATc1/αA (NCBI number NP_765978). Dominant negative inhibitor of Cullin1 were amplified from cDNA library with forward primer (5’-CGGGATCCGCCACCATGTCGTCAACCCGGAGCC-3’) and reverses primer (5’-CCGCTCGAGGACAACCATCACTTGATTGAG-3’) and cloned into pcDNA4mychis (A) to generate pCullin1-DN. Dominant negative inhibitor of Cullin2 pCullin2-DN were amplified with (5’-CGGGATCCGCCACCATGTCTTTGAAACCAAGAG-3’) and (5’-CCGCTCGAGGTCATCAATGTATTTGAAC-3’) and cloned into pcDNA4mychis(A). NFATc1-1-433, NFATc1-1-308, NFATc1-1-272 were amplified from p- NFATc1mycFLAG with T7 primer (5’-TAATACGACTCACTATAGGG-3’) and reverse primers, 5’-ccgctcgagCTGCACCTCAATCCGAAG-3’, 5’-ccgctcgag GGTGTACTGGGTGGTGTT-3’, 5’-ccgctcgag GCCGTTGAGGCTGTACTT-3’, and cloned into pCMV-mycFLAG. NFATc1-424-716 and NFATc1-307-716 were amplified from p- NFATc1mycFLAG with forward primers, 5’-cgcggatccgccaccatgCCGTATGAGCTTCGGATT-3’ and 5’-cgcggatccgccaccatg TACACCAGCTCGGCCAT-3’, and XL39 primer 5’-ATTAGGACAAGGCTGGTGGG-3’. S261Awas made by PCR site directed mutagenesis using two primers 5’-CTCCAGACCCGCGGCCCTTGCAACAAG-3’ and 5’-CTTGTTGCAAGGGGCCGCGGGTCTGGAG-3’. S278A was made by PCR site directed mutagenesis using two primers 5’-CAGCCGCCCTACGCACCCCACCACTC-3’ and 5’-GAGTGGTGGGGTGCGTAGGGCGGCTG-3’. S403A was made by PCR site directed mutagenesis using two primers 5’-AGCCCAAGCCCCTGGCCCCTACGTCCTACATG-3’ and 5’-CATGTAGGACGTAGGGGCCAGGGGCTTGGGCT-3’. S409A was made by PCR site directed mutagenesis using two primers 5’-ACGTCCTACATGGCCCCGACCCTGCCCGCCCTGG-3’ and 5’- CCAGGGCGGGCAGGGTCGGGGCCATGTAGGACGT -3’.

### Immunofluorescence

Immunofluorescence was performed as previously described [18]. Mouse anti-ubiquitin monoclonal antibody (3936; CST, Danvers, MA, USA) and cy3 labelledgoat anti-mouse IgG (715-167-003; Jacksonimmun, Fort Lauderadale, LF, USA) were used to detect ubiquitin. Images were captured using a Zeiss Microscopy LSM 780 fluorescent microscope and analyzed with Image J software.

### Western blot, co-immunoprecipitation and antibodies

Western blot and co-IP were performed as previously described [18]. Mouse anti-FLAG monoclonal antibody M2 (F1804; Sigma-Aldrich, Louis, MO, USA) and rabbit anti-FLAG polyclonal antibody (SAB4301135; Sigma-Aldrich, Louis, MO, USA) were used to detect exogenous NFATc1. Mouse anti-NFATc1 monoclonal antibody (MA3-024; Pierce, Rockford, IL, USA) was used to detect NFATc1. Mouse anti-HA tag monoclonal antibody (MMS-101R; Convance, IL, USA) was used to detect exogenous NFATc2. Mouse anti-ubiquitin monoclonal antibody (3936; CST, Danvers, MA, USA) was used to detect ubiquitination. Mouse anti-pSer monoclonal antibody (sc-81514; Santa Cruz, CA, USA) was used to detect serine phosphorylation. Mouse anti-β-actin monoclonal antibody (A1978; Sigma-Aldrich, Louis, MO, USA) was used to detect β-actin as loading control. Mouse anti-TBP monoclonal antibody (T1827; Sigma-Aldrich, Louis, MO, USA) was used as nuclear protein loading control. Mouse anti-DYRK1A monoclonal antibody (WH001859M1; Sigma-Aldrich, Louis, MO, USA) was used to detect total DYRK1A, according to the manufacturers’ instructions.IRDye 680 Goat Anti-Rabbit IgG(c10207-01) and IRDye 800CWGoat Anti-Mouse IgG (C11026-03) from Li-COR Biosciences (Lincoln, NE, USA) were used as secondary antibodies. Detection and quantification were performed with the Li-COR Odyssey imaging system.
